# Smartphone Users’ Persuasion Knowledge in the Context of Consumer mHealth Apps: Qualitative Study

**DOI:** 10.2196/16518

**Published:** 2021-04-13

**Authors:** Eunsin Joo, Anastasia Kononova, Shaheen Kanthawala, Wei Peng, Shelia Cotten

**Affiliations:** 1 Department of Public Relations and Advertising Beijing Normal University-Hong Kong Baptist University United International College Zhuhai China; 2 Department of Advertising and Public Relations Michigan State University East Lansing, MI United States; 3 Department of Journalism and Creative Media University of Alabama Tuscaloosa, AL United States; 4 Department of Media and Information Michigan State University East Lansing, MI United States; 5 Department of Sociology, Anthropology, and Criminal Justice Clemson University Clemson, SC United States; 6 Department of Communication Clemson University Clemson, SC United States

**Keywords:** mHealth app, personal health information sharing, mobile phone, mobile promotion strategy, persuasion knowledge

## Abstract

**Background:**

Persuasion knowledge, commonly referred to as advertising literacy, is a cognitive dimension that embraces recognition of advertising, its source and audience, and understanding of advertisers’ persuasive and selling intents as well as tactics. There is little understanding of users’ awareness of organizations that develop or sponsor mobile health (mHealth) apps, especially in light of personal data privacy. Persuasion knowledge or recognition of a supporting organization’s presence, characteristics, competencies, intents, and persuasion tactics are crucial to investigate because app users have the right to know about entities that support apps and make informed decisions about app usage. The abundance of free consumer mHealth apps, especially those in the area of fitness, often makes it difficult for users to identify apps’ dual purposes, which may be related to not only helping the public manage health but also promoting the supporting organization itself and collecting users’ information for further consumer targeting by third parties.

**Objective:**

This study aims to investigate smartphone users’ awareness of mHealth apps’ affiliations with 3 different types of supporting organizations (commercial, government, and nonprofit); differences in users’ persuasion knowledge and mHealth app quality and credibility evaluations related to each of the 3 organization types; and users’ coping mechanisms for dealing with personal information management within consumer mHealth apps.

**Methods:**

In-depth semistructured interviews were conducted with 25 smartphone users from a local community in midwestern United States. Interviews were thematically analyzed using inductive and deductive approaches.

**Results:**

Participants indicated that their awareness of and interest in mHealth app–supporting organizations were secondary to the app’s health management functions. After being probed, participants showed a high level of persuasion knowledge regarding the types of app-supporting organizations and their promotional intents. They thought that commercial companies sponsored mHealth apps mostly as entertainment tools, whereas noncommercial entities sponsored mHealth apps for users’ education. They assigned self-promotional motives to commercial organizations; however, they associated commercial mHealth apps with good quality and functioning. Noncommercial entities were perceived as more credible. Participants were concerned about losing control over personal information within mHealth apps supported by different organizations. They used alternative digital identities to protect themselves from privacy invasion and advertising spam. They were willing to trade some personal information for high-quality commercial mHealth apps. There was a sense of fatalism in discussing privacy risks linked to mHealth app usage, and some participants did not perceive the risks to be serious.

**Conclusions:**

The discussion of and recommendations for the safe and ethical use of mHealth apps associated with organizations’ promotional strategies and personal data protection are provided to ensure users’ awareness of and enhanced control over digitalized personal information flows. The theoretical implications are discussed in the context of the Persuasion Knowledge Model and dual-processing theories.

## Introduction

### Background

In today’s mobile-driven era, mobile apps have a significant impact on users’ healthy lifestyles [1]. Using mobile app services can help individuals manage chronic diseases and healthy lifestyles as well as fight bad habits, such as smoking [2,3]. Commercial, governmental, and nonprofit organizations support mobile health (mHealth) apps in diverse formats, and many do it with a dual purpose: to help improve public health and to promote their organizations [4]. For example, Under Armour sponsored and later purchased MyFitnessPal, a leading free app for achieving and maintaining health and fitness goals [5,6]. The Baby Center portal, formerly owned by Johnson & Johnson, and a corresponding pregnancy tracking app reached 45 million users in 2016 [7]. mHealth app support occurs not only in commercial sectors but also in public sectors, including nonprofit and government agencies. A US-based Centers for Disease Control and Prevention’s new parenting app, for example, offers customized services for parents to track their children’s developmental milestones [8]. The nonprofit organization the American Red Cross launched a blood donor app that enables the organization to communicate with its donors and offers a rewards program for users to increase the frequency of blood donations [9].

Despite the benefits to app users and app-supporting organizations, the growing consumer mHealth app market raises concerns over users’ awareness of app-supporting organizations and personal information privacy because personal data can be shared via mobile phones and wireless networks [2,10-13]. The mobile app market is dominated by free-to-use consumer apps, with more than 90% available without charge in the major app stores (ie, Apple App Store and Google Play) [14]. Instead of paying for such apps with money, mobile users may be asked to give away bits of personal information needed for an app to function. Personal information (eg, age, gender, and email), including basic health information (eg, weight and height), can be shared with unauthorized third parties via mobile phones and users do not easily recognize the magnitude of such information sharing [15-20].

Access to users’ personal information becomes a concern because mHealth apps’ affiliations with supporting organizations, especially those in the commercial sector, are often obscure [7]. This adds complexity to users’ interpretations of organizations’ motivations to support these apps. On the one hand, such support is driven by the intent to help users manage their health and help others. On the other hand, supporting organizations gain direct access to consumers’ personal data and may use these data for commercial, marketing, and other self-serving organization-related purposes (eg, fundraising or customized advertising). This emphasizes the value of studying users’ critical assessment of mHealth apps associated with different types of organizations, users’ knowledge of organization types and intentions to support mHealth apps, and users’ strategies to cope with privacy-related risks when they are asked to share personal information via smartphones.

This study contributes to the existing literature on persuasion knowledge and mHealth apps that serve the dual purpose of improving public health and promoting supporting organizations. Previous mHealth research has examined app selection process in app stores and issues related to potential privacy risks of personal information collection and management [3,10,21]. However, there is little understanding of whether and how smartphone users react to cues about organizations that support mHealth apps and how they understand and negotiate the duality of organizations’ motivations (ie, users’ health management vs organization self-promotion). Furthermore, few studies have explored differences in users’ perceptions of mHealth app quality and credibility and willingness to share personal information across organization types: commercial, government, and nonprofit, in light of organizations’ dual motivations to support such apps. To address these gaps, this study applies the Persuasion Knowledge Model (PKM), dual-processing theories, and information privacy literature to explore how mHealth apps associated with commercial, government, and nonprofit organizations influence smartphone users’ understanding of mHealth apps. It investigates mHealth app use not only as a health management tool but also as a promotional tactic. The study provides useful insights about mHealth app cues associated with commercial and noncommercial organizations that can be used to efficiently communicate mHealth app affiliation to users and enhance their critical assessment of this health-related technology. This study also provides suggestions for government and nonprofit organizations to offer effective and engaging mHealth app services that ensure individual autonomy in protecting personal data.

### mHealth App Market

The mHealth app market was valued at approximately US $12.4 billion in 2018 and is expected to expand at a compound annual growth rate of 44.7% from 2019 to 2026 [22]. The World Health Organization broadly refers to mHealth as “medical and public health practice supported by mobile devices, such as mobile phones, patient monitoring devices, personal digital assistants (PDAs), and other wireless devices” [23]. Some mHealth apps are provided by medical organizations to communicate personal health information (PHI; eg, medical test results, prescriptions, and diagnosis) to patients. Such apps fall under the category of personal health records. They provide a single space for patients to access their own health records and simplify patient-provider communication [24]. Alternatively, many mHealth apps can be downloaded from app stores without providers’ involvement in the user’s initiative to maintain a healthy lifestyle or to enhance the health of others [25]. These apps are often developed or owned by companies with aligned stakes, such as MyFitnessPal, or standalone health-related apps, such as Flo, the period tracking app. The information provided to the app comes from the user based on their own knowledge of their health and data tracked based on app usage. Such apps have no direct connection to health professionals.

Today, almost 60% of US smartphone users have downloaded at least one health-related app on their mobile devices [26], with the exercise and weight loss app category being the most popular [3,4]. It is common for users to provide personal information, including basic health information (eg, height, weight, BMI, physical activity levels, calorie and water intake, pregnancy status, sleeping patterns, mood, period, and sexual activity), for customization by direct input or connecting to sensors and wearable technologies [4,5,10]. This information is typically less protected, especially if mHealth apps are free to use. Sharing users’ data with digital marketers within and outside the organization becomes a source of the sustainability of these apps [2,18]. This study explores smartphone users’ perceptions of these consumer mHealth apps, with the focus on persuasion knowledge and privacy concerns.

### PKM and Promotional mHealth Apps

Persuasion knowledge is important for users with respect to mHealth apps because they are created for users’ health management and organizations’ promotional purposes. Conceptually, persuasion knowledge, commonly referred to as advertising literacy, is a cognitive dimension that embraces recognition of advertising, its source and audience, and understanding of advertisers’ persuasive and selling intents as well as tactics [27]. Conceptual persuasion knowledge is different from evaluative persuasion knowledge that deals with consumers’ affective evaluations of advertising. According to the PKM, people develop and use their knowledge derived from previous experience, education, and socialization to recognize, interpret, evaluate, and respond to persuasion attempts, such as advertising [28]. As a reaction to persuasion (promotion) attempts, media users choose and execute persuasion coping behaviors that they perceive as effective and appropriate. Such behaviors may be positive for a brand or organization that promotes itself using an mHealth app (eg, buying and telling others about the organization or app) and may be negative (eg, deleting the app and boycotting the organization) [29]. In the contexts of brand-related apps, the more favorable users feel about a brand-supported technology, the more receptive they are to its claims or content, including the identification of a supporting organization [30,31].

The PKM includes 3 belief structures [28]. The first structure refers to persuasion knowledge itself, where consumers are aware of and understand actors in the self-promotion persuasion process, persuaders’ intentions, and tactics used to persuade, among others. In relation to promotional mHealth apps, persuasion knowledge may refer to app developers and sponsors who put the app on the market, understanding the reasons for app support and perceptions of the app as a promotional tool to increase sales, donations, public awareness, and other desirable outcomes. The second structure is related to the perceptions of the persuasion agent (eg, advertiser), including the agent’s traits, competencies, and goals. In the context of this study, we discuss 3 types of persuaders: commercial, governmental, and nonprofit entities. Understanding the nature of each persuader, its resource base, and mission constitutes beliefs about the agent. The third structure is associated with beliefs about the topic of persuasion (eg, product, service, social cause, or candidate). It could be, for example, related to a specific health issue that an app focuses on (eg, fitness and diabetes) and the digital app itself (eg, MyFitnessPal).

When consumers’ persuasion knowledge levels are high, they are able to understand the self-promotional intent and, as a result, doubt the altruistic intentions of an organization and activate cognitive defenses against persuasion [21,32,33]. Consumers with high persuasion knowledge are more likely to be skeptical of self-promotion persuasive communication and resistant to persuasive advertising messages or sponsored products [29,34]. Persuasion knowledge plays a significant role in evaluating subtle (vs prominent) digital internet-based advertising formats [29,34]. For example, users with a higher level of persuasion knowledge may be more likely to recognize a supporting organization when they examine an mHealth app (especially if such an app is offered for free) and form their own beliefs about the organizations’ intentions, be they for the public good (eg, social responsibility and establishing healthy lifestyles) or self-service (eg, profit seeking, data collection and sharing with third parties, and social control).

When evaluating an organization’s support of consumer technology, such as mHealth apps, and making usage decisions, users may negotiate between organizations’ self-promoting and user-oriented motives differently across different types of app providers, especially if they are asked to give away personal information to download and use the app [12,16,35,36]. Previous studies have indicated that nonprofit sponsors of information and communication technologies receive more favorable evaluations than their for-profit counterparts. This is reflected in the positive attitudes and attribution of less egoistic motives to nonprofit entities [36,37]. Although consumers may have difficulty inferring selfish motives in cases of prosocial persuasion attempts, such as antidrunk driving campaigns (or, in the case of this study, public health), they would still trust nonprofit or government agencies as campaign sponsors more than corporate organizations [37]. We suggest and further explore the type of organization associated with different degrees of negotiation between self-serving and public service motives when smartphone users decide to download an mHealth app and share personal information within it. This study aims to examine the nature of persuasion knowledge and mHealth app evaluations associated with 3 types of app-supporting organizations (ie, commercial, governmental, and nonprofit) and to determine if willingness to share information within mHealth apps differs by organization type.

### Sharing Personal Information via mHealth Apps

Many smartphone apps require users to give permission to access personal data (eg, social media data and contact lists) and phone functions (eg, camera and speakers), and such permission is very easy to obtain (eg, clicking “I Agree”). As a result, issues related to sharing personal information with the app are emerging as an important research area for health practitioners and policy makers [10-12,19,20]. Personal information obtained via the apps can be shared within the supporting organization itself as well as with unknown third parties, such as marketers, without explicitly notifying app users [15-20]. At the initial level, users might not realize that the app they use is affiliated with an organization. For example, formerly Johnson & Johnson’s Baby Center portal and app used to have only subtle cues (eg, pop-up advertisements) about such affiliation. Furthermore, users may not always realize that the personal information they provide is used within and outside the company for marketing purposes. This may facilitate the risks from emotional distress to financial discrimination. For example, period and pregnancy tracking consumer apps are popular not only with female smartphone users but also with companies, such as Johnson & Johnson, which target this demographic with offers of women- and parenthood-related health products. Users share sensitive health information with such apps, including patterns of sexual activity, number of pregnancies and miscarriages, and gestational age of unborn children. Mere awareness of such data being shared for marketing purposes may create psychological discomfort. Moreover, other companies may be interested in accessing this target group’s data to promote relevant products (eg, cars and realtor services). Although the data are deidentified and aggregated when moving through a complex analytics process, they help profile individuals and assess their qualifications for life insurance, mortgages, and loans [38-40].

The classical definition of privacy is the freedom to protect oneself from exposure to or intrusion by others [11,41]. Privacy is an important requirement in the health care domain that deals with the challenges of maintaining PHI about one’s condition and health history confidential while sharing them with authorized medical parties and caregivers, guardians, and family members [42]. Although strict rules apply to protecting one’s PHI [2], policies to manage one’s personal information (including basic health information) provided to consumer mHealth apps are less clear.

In the digital age, this is discussed in the context of having autonomy. Autonomy refers to an individual’s right to control the environment in which they live in and make rational decisions [43]. An mHealth user, for example, can decide to download or delete the app at any time; thus, they have control over using it. However, can they make such decisions about personal and health data collected via this app? Can they recognize persuasion attempts when they see marketing messages tailored to them using sophisticated technologies? Privacy is described as a tool that fosters and encourages autonomy. Becker [43] argues that omnipresent digital technologies that allow constant surveillance put at risk not only privacy—when an individual is *observed* on the web and *analyzed* via algorithms—but also autonomy where the individual loses the power to make informed decisions about their personal information flows (especially when information is deidentified and aggregated) as well as make independent decisions about customized marketing messages.

Some users avoid downloading mHealth apps because of privacy concerns and potential risks related to the collection of personal identifiable information [3,10,35]. Users also consider advertising messages and commercial identification (eg, brand logo) as negative cues when making credibility judgments of sponsored websites [36,37]. According to the literature on dual-processing theories and decision-making processes, users tend to rely on heuristic cues to simplify the selection process if they face cognitive limitations because of a deluge of information [11,35,44]. Such heuristic-based decision-making processes may promote automatic app judgment and selection that does not require much effort for a thorough app evaluation [11,45]. Users, for example, rarely read app privacy policies; that is, they rarely engage in systematic, elaborate, and effortful information processing before downloading an app unless they are first informed about negative consequences of sharing personal information [11,18,19]. Instead, they relied on app visual cues to assess it [45]. Many do not worry, are unaware of data sharing with third parties, or cannot imagine the large scope of such sharing [13,19].

Sources of digital content (eg, developer, owner, or sponsor in the context of mHealth apps), if visible and recognizable, may serve as a powerful cue to activate heuristics that guide the evaluation of the digital content [35-37]. These heuristics may differ according to the type of source. In this study, we suggest that smartphone users have different mental representations of commercial, governmental, and nonprofit organizations that support mHealth apps. Such differences manifest themselves in unique perceptions of each organization type, including its characteristics, capacities, and self-promotion versus public service motivations, differences in assessing the quality and credibility of mHealth apps associated with different types of supporting organizations, and different levels of willingness to share PHI with supported mHealth apps. Understanding these differences will inform the development of transparent mHealth technologies that effectively communicate information about technology affiliation with an organization and equip users with strategies to protect their personal information when engaged with technology use.

### Research Questions

Applying the PKM [28], dual-processing models [11,44,46], and the concepts of information privacy discussed earlier, we ask the following research questions (RQs):

- RQ1: What are study participants’ levels of awareness of and interest in knowing an mHealth app affiliation with a supporting organization?
- RQ2: What are the differences in the nature of participants’ persuasion knowledge (eg, recognition of target and agent and understanding of agent’s characteristics and capacities, self-promotion, and public service purposes) across commercial, governmental, and nonprofit organizations that support mHealth apps?
- RQ3: In what ways do participants’ evaluations of mHealth apps’ quality and credibility differ by the type of supporting mHealth app organization?
- RQ4: What are the differences in coping mechanisms, if any, that participants implement when they are informed about mHealth app support by commercial, governmental, and nonprofit organizations, especially in light of sharing personal information within such apps?

## Methods

### Recruitment

In-depth semistructured interviews were conducted at a large university in the midwestern region of the United States. The study protocols were approved by the institutional review board before data collection began. Local community residents (N=25) were recruited through a web-based recruitment pool. Each participant received US $15 for their participation in the research. Data collection was stopped at a sample size of 25 because of response saturation. Specifically, the last participants interviewed confirmed the responses from earlier interviews and did not provide additional novel insights [47,48].

### Procedure

The average time for each interview was 52 minutes, ranging from 36 to 92 minutes. All interviews were recorded using digital recording options available on the interviewers’ smartphones. A total of 3 researchers trained 2 student interviewers and oversaw the data collection process. Participants were informed about being recorded in the consent document before they started the interview. All participants had the option of withdrawing from the study or refusing to answer any question; none of them did. The full interview guide is presented in Multimedia Appendix 1. Participants were asked about their perceptions of *the government*, *commercial*, and *nonprofit* apps. Examples were provided per the participants’ request. Most of our participants were familiar with the concept of mHealth apps and did not need an explanation as to what health apps were.

### Data Analysis

Anonymized interview audio files were transcribed using a web-based transcription service. Data coding was continued with 4 coders (coauthors). As per standards of qualitative methodology and, specifically, thematic analysis [49], the coders were open in their approaches to raw data analysis and flexible in revising transcript interpretations. NVivo, a qualitative data analysis computer software, was used to organize the codes and corresponding data. To assess coders’ agreement with the generated codes, 3 identical transcripts were independently analyzed. Coders met multiple times to discuss emerging codes. The coding tactics were refined iteratively in each meeting. First, coders discussed each code they identified, both on descriptive and interpretative levels [50], and unified names and definitions of codes that they agreed upon. After the initial rules of coding were established based on multiple readings of the same transcripts and each code received a clear definition, 2 coders coded the remainder of the transcripts. A total of 139 codes and subcodes were developed with 538 references (Table S1 in Multimedia Appendix 2). The codes and subcodes were then analyzed to inductively derive major themes [51].

We chose to base our coding on empirical data collected more than on previous literature (ie, inductive approach) because of the centrality of organization type to the conversation between participants and interviewers. As little work, to our knowledge, has been done about the perceptions of mHealth apps supported by government, commercial, and nonprofit organizations, we did not impose a strict top-down structure (deductive approach) on the coding rubric. A deductive approach was used after the themes were derived to organize our reporting of the findings in accordance with the theoretical frameworks used and the RQs examined.

To ensure the trustworthiness and consistency of the findings, we used a number of procedures [48,49,52]. These included team-based instrument development designed to achieve the study’s objectives, extensive training of interviewers and coders, data collection oversight, using multiple coders to work with transcripts, developing a coding rubric and establishing consistent analysis routines (regular discussions of code and theme interpretations), applying logic to assess code relevance and irrelevance, and supporting themes with quotations from participants.

## Results

### Overview

The participants’ demographic descriptions are provided in Table S2 (Multimedia Appendix 3). The findings provide descriptive information about the participants’ use of smartphones, apps, and mHealth apps and report themes derived through the analysis (Multimedia Appendix 2).

### Mobile Phone and App Use: Descriptive Information

More than half of the interviewees had an iPhone (13/25, 52%), 6 had a Samsung smartphone, and 6 had other types of smartphones. The average length of smartphone use in the sample was approximately 3 years. On average, interviewees had 29-32 apps on their phones. The most used apps were social media apps (Facebook, Instagram, Snapchat, etc). Other frequently used app categories included email, messaging, video chatting (eg, Skype), utility (eg, weather and maps), game, shopping, banking, entertainment streaming (eg, Netflix and Spotify), and health and fitness (eg, MyFitnessPal) apps. In total, 9 of the 25 (36%) participants reported that they had at least one health-related app on their smartphones. Most health-related apps mentioned by the interviewees were related to maintaining healthy lifestyles (eg, step counter, calorie counter, running tracker, healthy eating, and meditation), and 2 mentioned disease or disorder management apps (eg, attention deficit hyperactivity disorder).

### RQ1 and RQ2 Findings

RQ1 asked about study participants’ levels of awareness of and interest in knowing an mHealth app affiliation with supporting organizations.

#### Recognition of mHealth App Source Is Secondary to the App’s Health Management Functions

Participants expressed little awareness of and curiosity about promotional app support before we probed them with questions related to the study’s RQs. Most participants said that they rarely paid attention to information about mHealth app developers, sources, and sponsors. For them, mHealth apps’ ease of use, information quality, relevance, and functionality or utility were more important than an entity supporting the apps:

If it records what I want, I do not care who designed it. If it’s easy to use and if it has all the information I need, then that’s why I would do it.

Participant 9, female, aged 65 years

Many participants elaborated that any mobile app was easy to delete; thus, apps did not pose any danger and did not lead to users’ personal data breaches. For example, one participant (participant 14, female, aged 29 years) indicated that the use of promotional mHealth apps was not a *serious* issue. Thus, at the initial level of discussing promotional mHealth apps, users were consumed with the health management function of these apps and did not consider additional, self-promoting motivations that could drive the organization’s app support.

RQ2 asked about the differences in the nature of participants’ persuasion knowledge across commercial, governmental, and nonprofit organizations that support mHealth apps. After being probed, most interviewees indicated that they possessed persuasion knowledge of app-supporting organizations. They perceived themselves and similar groups of consumers as well as society as a whole to be the targets of persuasive attempts initiated mostly by commercial companies.

#### From Commercial Entertainment to Noncommercial Information

Discussing the characteristics and capacities of organization types, participants were more likely to assign *general information* function to government agencies. Overall, increasing public awareness of health problems and educating people about them was the overarching goal of governmental and nonprofit agencies and associations. As for commercial companies, their products and services, while associated with the best quality, were mostly perceived as *fun* and *entertaining*:

Bono’s probably on there [commercially supported mHealth apps] talking or something. They’ve probably got a quote from Jay-Z on there.

Participant 23, male, aged 25 years

Government agencies were perceived as being research driven and resourceful in terms of the health information available. As the government’s role in supporting mHealth apps was mostly discussed as being a general, broad information provider, its mHealth apps were perceived as secondary to the web resources that provided a great deal of credible medical information to participants. It was easier for participants to access these web resources on their computers, rather than via mHealth apps:

If I wanted to search Metformin, diabetic drug, then everything that’s on the web on that...

Participant 9, female, aged 65 years

I’ll search stuff like that online, on the Internet, but I don’t need an app for it...

Participant 24, female, aged 24 years

Narrow specialization, knowledge of one problematic health area, was the prerogative of nonprofit organizations:

Their goal is to provide the health information or the healthcare information that is needed by the user without focusing on other areas that the person doesn’t need. If I have to find out about cancer, I’m [going to] go to the American Cancer Society app, I’m not [going to] go to the one about diabetes.

Participant 10, male, aged 65 years

#### Self-Promotion and Public Service Motivations Behind mHealth Apps

Participants recognized strong self-promotion intentions of for-profit organizations to support mHealth apps, such as selling products, advertising a company, and building brand image. Some explained that commercial companies used apps to misinform users and collect consumers’ *opinions*, record personal information, and track app usage data. Only a few attributed commercial app support to corporate social responsibility oriented to public service:

That’s like an advertising platform for them. So, if you were to get an app to count calories while you were running, that app’s created by Nike, at some point it would try to sell you some kind of Nike shoes. So yeah, it’s an advertising platform for commercial organizations besides, of course, getting some consumer goodwill.

Participant 11, male, aged 40 years

Although participants could clearly distinguish between the intentions of commercial and noncommercial organizations, there was little understanding of the differences between the government and nonprofit sectors. Participants expressed great concern about paying *my money* for commercial products and services but they were much less worried about how taxpayers’ and donors’ money was spent by government and nonprofit organizations. They perceived nonprofit and government agencies to be more careful and accountable in spending, especially that these agencies, according to the participants, had a clear goal of improving public health by helping people via mHealth apps. Thus, public-serving motivation was more pronounced in participants’ perceptions of noncommercial entities. Only a few participants mentioned that nonprofit and government organizations developed mHealth apps for self-profiting reasons: to collect personal information and to fundraise for a cause.

The topic of trust in an organization’s public service motivation was related to the discussion of congruency between an app-supporting organization and the health issue or cause. Interviewees expressed distrust in some commercial organizations that would support an irrelevant health issue. One participant called it *hypocritical*. Congruency was identified not only on the level of the cause but also on the level of the organization’s general mission. Interviewees found that it was more relevant for nonprofit organizations to support health apps because it was consistent with their goals of making people healthier and contributing to the overall social good:

I would always pick the government organization or the non profit organization, because I feel they are doing it for the public good, because they don’t have anything to gain by it, as opposed to somebody [commercial companies] who is doing it...to make money off of it.

Participant 20, female, aged 54 years

### RQ3 and RQ4 Findings

RQ3 asked how different types of supporting mHealth app organizations influenced participants’ evaluations of mHealth apps’ quality and credibility.

#### Quality Does Not Mean Credibility

The discussion of advertising literacy and mHealth app evaluations centered on 2 aspects of app evaluation: perceived app quality and perceived app credibility. We left the definitions of these constructs open so that participants interpreted them according to their definitions of *quality* and *credibility*. Overwhelmingly, participants discussed mHealth app *quality* as related to the apps’ looks, usability, and functionality while they talked about *credibility* as related to trust and intentions of supporting organizations:

I expect [commercially supported app] to look fancier and brighter. I will say the quality to be better as in its functionality, it shouldn’t have any slowness when you download it, type thing. Not actually having more options, just its functionality should be better from big companies...I don’t put much credibility into their things [commercial companies] because a lot of things they do are for profit...I like non profits, I would do something from American Cancer Society probably easier than I would accept something from Coca-Cola.

Participant 13, female, aged 23 years

Perceptions of organizational resourcefulness were directly linked to mHealth quality judgments. A distinguishing feature of quality was related to *dumping more money* into app development. Commercial organizations were perceived as more willing to invest in better quality apps compared with *less rich* nonprofit organizations or government agencies. There was no expectation for publicly (not commercially) supported apps to look appealing:

They [nonprofit and government organizations] might get the job done, they might capture the information, but it’s not going to keep my interest, or be visually appealing, or have the functionality that I need. And so, I still wouldn’t pay for it though, but I may have to look at a [commercially] sponsored app.

Participant 2, female, aged 48 years

A prominent perceived characteristic of government-backed apps was related to providing scientific, research-supported health information and help for people:

I would give credibility to the Health and Human Services or a Center for Disease Control. It all depends on the particular information I’m looking for, whether it’s about preventing the spread of a disease, or if it’s developing good practices for personal health care. The government has a place for its information.

Participant 10, male, 65 years

Another valuable characteristic of government-based mHealth apps was security. With this came trust in government-supported digital products:

I would trust more in a governmental app in this case, just because they have more rules about security and it’s probably harder to get access to governmental data.

Participant 21, anonymous

Overall, participants described commercial organizations as less credible because self-promotion and profiting were perceived as the obvious drivers of mHealth app support. Nonprofit organizations and government agencies were described as more trustworthy than their commercial counterparts. However, some participants mentioned the complexity of indicating true motives in relation to all 3 organization types because they supported mHealth apps not only *to make people healthier* but also for publicity and control.

Despite a clear understanding of commercial organizations’ intentions, participants leaned toward using commercially supported mHealth apps, as they were better in appearance and features. In other words, they valued app quality more than information credibility. Few participants were willing to *sacrifice* visual appeal and functionality for more *ethically* supported and credible mHealth apps from noncommercial sources.

RQ4 asked about the differences in coping mechanisms, including the strategies to protect personal information shared electronically, that participants implemented when they were informed about mHealth app support by commercial, governmental, and nonprofit organizations.

#### Trade-Offs

Most participants, while having privacy concerns, did not have the habit of reading privacy terms and conditions before downloading and using mHealth apps. Instead, they used social and heuristic cues, such as star ratings and user reviews, to make downloading decisions. Only one participant claimed that he had read app privacy policies.

Many agreed to *pay* for mHealth apps use with their personal information and increased risk of this information being used for purposes not related to app functioning:

As long as it is not my social security number and my credit card number, I tend to give that information [to app supporting organizations]. It might be a win-win. I mean it might also help me manage my health but of course the commercial for-profit companies are always interested in my opinion, and always interested in making sure that you buy their product or are aware of their product.

Participant 2, female, aged 48 years

When participants had to share personal information, they indicated that they preferred to share less information than more information and general information over specific medical information:

I would be willing to give more general types of information, as opposed to very specific information, like, “Do you take 150 micrograms of Lexo, whatever, a day?” I would not [want to] say that’s what I do, but I would say, I’m on a thyroid medication.

Participant 20, female, aged 54 years

Furthermore, participants tended to agree to share greater amounts of personal information with nonprofit or governmental organizations than commercial organizations to use mHealth apps because they believed that nonprofit and government agencies used such information for public health and research:

I’m going to probably choose something that is either government or non profit related before I would choose something that’s for-profit before, just because I am assuming that for-profit it’s probably [going to] want more of my information to try to get me to purchase their products.

Participant 8, female, aged 36 years

Many participants were open to providing some personal information to download and use mHealth apps:

It doesn’t bother me, I think I’m not a superstar.

Participant 14, female, aged 29 years

They shared basic personal and health information, but it depended on how much information the app needed them to share and whether this information was relevant to the purposes of the app. Demographic information that participants were willing to share commonly included name, email, and date of birth. Interviewees shared health information if they were relevant to basic app functioning. There were also some differences in the type of information that could be shared with apps provided by companies, nonprofits, or government agencies:

If I have to put in my personal information, it depends on how much personal information. In order to use the [mHealth] app, I have to tell them [app developers and supporters] how old I am and how much I weigh and how tall I am and those kind of [things].

Participant 4, female, aged 34 years

I avoid trying to give out my postal address because most times in commercial, it’s just gonna give you junk mail...My weight, my height are fine...Depending who the non profit organization, I very well may give more information than a commercial...I wouldn’t give them like the lab paperwork, but I would be more interested in saying, “Hey, this is what may or may not be going on with me and this is what the kind of information I’m willing to give you about myself.” So, I feel that non profits can get a little bit more information because they’re not in it for the money.

Participant 7, male, aged 28 years

In addition, some participants suggested that signing in with an existing account on a social networking site, such as Facebook, was an easier way to download and use an mHealth app rather than creating a new, app-specific log-in. Participants did not see this type of information sharing as a threat to their privacy, as they did not consider personal information shared on social media to be private:

Yeah, I do that, I’ll connect, say that way they can, I guess I give them some of the information. Because if I’m posting on Facebook, I feel that’s generally public information amongst everyone.

Participant 7, male, aged 28 years

Convenience was the key reason for using social media sign-ins instead of creating an app-specific password. It was, as few expressed, a good way to save time and energy put into registering and accessing a new mHealth service.

#### It Is Culture and It Is Normal

Some interviewees expressed a feeling of fatalism and helplessness when it came to using promotional apps and sharing personal data with the supporting organizations and third parties. One participant, for example, understood the scope of the issue; yet, the issue was systemic and too big for one individual to combat:

I think I just assume that anything that I input into an app or put online, it could be seen by someone, or by a company, or whatever. Someone could be tracking it and so, I don’t think I necessarily put too much thought into privacy, other than with the Mood Tracker app that I was looking at where you could choose to pay more and have it be private so it looked like other users won’t be able to see your data, but the company can still see your data. I guess I feel like there’s not really options of...No matter what app you have, somebody’s [going to] be able to see it, somebody’s [going to] be able to track your data and so it’s just the risk you take I guess in doing that.

Participant 12, female, aged 24 years

Another participant, an international resident of the local community, looked at this overpowering phenomenon as a consequence of the culture of transparency. As it was part of the culture, it was normalized to the participant’s mind:

I think I’m already kind of used to this culture here that, because if you go to see dentist, you’re, also when you’re providing any information about what happened to you. That’s very important, like you watch, kind of, you’re alert to what kind of things. That’s very normal, so you need to provide that.

Participant 14, female, aged 29 years

#### Defenses Against mHealth Persuasion Attempts

Some participants indicated they were less willing or unwilling to share any type of personal records. One reason was the potential to raise security risks related to identity theft and financial losses. Another reason was linked to privacy invasion or sharing personal information that participants preferred to keep private or have control over:

I’m not a huge fan of the intrusion via apps [...] I guess I’m not comfortable with how much companies can learn about me without my permission.

Participant 6, female, aged 23 years

Advertisements, junk mail, and email from commercial companies contributed to another reason the interviewees were averse to providing personal information; they were worried about app providers using their information for commercial purposes rather than for the needs of the app. Commercial purposes were associated with traditional print and digital advertising, such as receiving direct mail and promotional emails (spam):

Yeah, I avoid trying to give out my postal address because most times in commercial, it’s just [going to] give you junk mail.

Participant 7, male, aged 28 years

I would be less likely to put that information to a commercial app than to a non profit app, because I would be wondering what they were [going to] do with that...It’s a good way of cheap advertising.

Participant 20, female, aged 54 years

Several participants admitted that they used alternative personal information, such as secondary email addresses or fake names, to sign in to mHealth apps and thus avoid potential privacy invasion by third parties. This finding suggests that technology users seek creative ways to deal with privacy breaching threats that do not require extensive mental work to go over pages of hard-to-read terms of use:

I would use a name and date of birth, but it’s [going to] be fake for me almost no matter what website I sign up for. When it’s just some app or some website I’m not as apt to do that, but I’ll give them a fake name. I don’t really like the date of birth, but I know that sometimes you have to verify age. I wouldn’t give an address really for any app.

Participant 18, male, aged 23 years

## Discussion

### Principal Findings

The results of the study showed that participants were admitted to not paying much attention to mHealth app–supporting entities. An interesting strategy identified through our research is related to the tricks that our interviewees used to *play the system*. While understanding persuasive intents and having concerns about personal information privacy, participants did not engage in reading terms of use when downloading mHealth apps. Instead, they tried to protect themselves by using fake names and email or social media accounts that were designated to receive *junk* advertising messages.

When probed, the participants recognized the intentions behind commercial support. Most participants possessed high levels of persuasion knowledge. They were more likely to assign selfish than altruistic motives to commercial companies that support mHealth apps. Participants believed that nonprofit and governmental organizations spent their funds with greater accountability and supported health apps to protect public health. Although they distinguished between the intentions of commercial and noncommercial organizations, there was a lack of clear distinction between government and nonprofit agencies.

Many participants agreed to provide personal information to download and use mHealth apps. However, some participants expressed concerns about doing it because of potential risks related to invasion of privacy, data security, junk mail, and possible misuse of information by a third party. The discussion of *annoying* advertising messages that target app users to sell products was much more prevalent than the concern of sharing personal information with unknown parties without user awareness and consent. Participants did not reveal in-depth knowledge of the personal information sharing process in the digital sphere (eg, the use of artificial intelligence in advertising, algorithmic ad delivery based on digital data clusters, and programmatic buying). This leads to an important conclusion that the consequence of personal information sharing, including basic health information, shall be experienced in a relatively direct way, where individuals make straightforward associations between information sharing and receiving unpleasant promotional messages.

When the discussion touched on more abstract topics related to privacy invasion and required an understanding of complex processes that support the practices of data sharing through mobile and wireless networking technologies, participants were less likely to be concerned. Some participants, however, were less likely or unlikely to share personal information with consumer mHealth apps, regardless of the type of source organization. These participants had the general idea of abstract dangers related to privacy breaching and used the strategy of *being on the safe side* by distrusting any mobile app data sharing requests. Some interviewees, even if they decided to provide personal information, as it is a common element of the contemporary app use culture, were still concerned about the misuse of their personal information that would serve organizations’ commercial interests instead of being used exclusively for the app’s direct purposes. This suggests that it is possible that participants might not entirely understand the mechanism of misuse but they are at least roughly aware of what is happening while using data sharing apps. These findings suggest that some foundational knowledge would be useful to empower them to purposefully engage with data sharing and to protect themselves from undesirable practices [53-55].

Another theme that emerged in this study is related to *trade-offs* or *paying* with personal information for consumer mHealth app services. Participants not only agreed to trade personal information to receive good quality apps but also perceived commercial companies as providers of better quality apps than nonprofit and government agencies. At the same time, participants expressed helplessness related to the overwhelming nature of the digital sphere. It was normal to share personal information; it was part of the culture. Few interviewees agreed to use less visually appealing and functionally convenient mHealth apps if they were supported by nonprofit organizations and government agencies. This finding suggests that smartphone users rank usability, functionality, and visual appeal much higher than the credibility of the health information provided, despite the potential sensitivity of such information. This finding is particularly important in understanding the coping strategies employed by mobile app users. Therefore, it is important to explore this topic in future studies.

The match between an organization and an app cause or topic mattered. Participants were more willing to share personal information with mHealth apps if they perceived supporting organizations to be relevant to a health issue. The match between a supporting organization and an app was found on the abstract level of the organization’s mission. Some participants expressed more enthusiasm downloading an mHealth app supported by a nonprofit organization, as it was believed to serve people’s interests related to public health. Although the mission of nonprofit organizations was often viewed as helping people, government agencies were perceived as instruments that provide credible, research-based, and unbiased information. These findings contribute to our understanding of how the type of persuasion knowledge agent can influence consumers’ perceptions of the agent’s traits, competencies, and goals.

### Theoretical and Practical Implications

Some study findings echoed the results of previous studies. For example, we found that participants did not engage in reading app use agreements and privacy policies [10,12]. Expecting users to read large amounts of privacy information for a simple service such as an mHealth app is unrealistic, as it requires time and effort [10-12]. Previous research has shown that users often engage in trade-offs between being concerned about privacy of personal data and the potential benefits of using new technologies [10-13,19,20], whereas other studies have also suggested that users’ decisions are simultaneously influenced by other factors, such as risk (privacy concern) and trust (perceived control) [56,57]. Similar to previous studies [12,16,35-37], this study showed that the majority of participants were less likely to trust a commercial organization than a nonprofit or governmental organization supporting an mHealth app.

To extend previous evidence, this study offers several novel findings. First, it explored the complexity of assigning self-promotion and public service motivations to the following different types of supporting organizations: commercial, governmental, and nonprofit. It applied the PKM theoretical framework to further our understanding of persuasion knowledge related to not only commercial companies but also noncommercial entities. The findings of this study will help government and nonprofit organizations to develop technology-based cues for smartphone users to make effortless, yet informed judgments about consumer mHealth app quality, credibility, and personal information protection. Such cues may involve an organization’s logo and sector identification (for-profit [US $], nonprofit [♡], and government [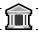]). Cues can be created to identify the nature and the scope of personal and health information sharing. For example, such data can be labeled as those that are (1) not shared outside the app, (2) shared within the supporting organization, and (3) shared with third parties. The purpose of sharing could also be specified via the following visual cues: marketing and promotional message personalization, public health statistics, or other.

It might still be unclear whether solely user-centric approaches will be effective in educating users about information privacy protection, as shown by our findings that extend the scope of existing literature [58]. Thus, we suggest that through relevant policy and grassroot efforts, leaders guarantee user autonomy and privacy when using consumer mHealth apps and increase the motivation of stakeholders to establish ethical rules of consumer mHealth app execution and personal data distribution for long-term success [59]. Initial efforts to voice concerns of mHealth users have recently been made by both nonprofit and government organizations. For instance, the Federal Trade Commission, in 2016, released guidelines about each app, requiring an explanation in plain language of the kind of data the app would collect, and who would have access to it [60]. In addition, a 2018 California law (with a compliance date of January 2020) that focuses on consumer apps that do not fall under the purview of the Health Insurance Portability and Accountability Act requires updated privacy policies and implementation of a consumer’s right to erasure [61,62]. Furthermore, Xcertia, a nonprofit organization founded by the American Medical Association and other major health and technology organizations, released guidelines for mHealth apps that would help with the privacy and security of users’ health information [63].

This study provides additional practical insights related to promotional mHealth apps. To build a positive brand-consumer relationship through new mobile communication channels, commercial companies should consider users’ persuasion knowledge and advertising literacy levels because awareness of the supporting organization’s true intentions, especially oriented toward serving the public with health management tools, may positively affect app and brand evaluations. From the findings of this study, we can conclude that commercial organizations specifically elicited mixed perceptions among participants. Commercially supported mHealth app quality was attractive to users; however, the primacy of self-promotional motives had negative connotations. It would be beneficial to continue the investigation of mixed attitudes toward commercial mHealth apps to identify situations when such apps are perceived as bad and misleading and when they are viewed in a positive light through users’ personal experiences. Furthermore, it is necessary to think more carefully about a good match between an organization’s focus and mission and an mHealth app topic. Otherwise, mHealth app promotional support could result in a negative perception of the supporting agency.

Although commercial support implies strong self-driven intentions and, thus, could elicit skepticism, government and nonprofit support could be associated with the goal of improving public health [37]. It is important to facilitate the support of mHealth apps by noncommercial organizations, as such organizations may leverage trust and perceptions of their digital products as highly credible, backed by research, and secure. Security, although not being the focus of the study, often emerged in participants’ discussion of privacy, supporting previous literature that shows the intertwined (objective and perceived) nature of the 2 concepts [64-66]. Future studies will need to determine how different types of supporting organizations would affect users’ perceptions of consumer mHealth apps at the same level of usability while also exploring the tipping point when the credibility of noncommercial apps becomes more appealing to users than the usability of commercial apps.

Finally, it may be difficult to fully distinguish between the 2 types of mHealth apps: those that are designed to enhance public health and those that collect personal and basic health information for marketing purposes. Promotional apps do not exclude the purpose of providing high-quality health services. Instead, they might be characterized by purpose duality, where an organization’s promotion happens by providing good mHealth services. The findings of this study indicate that purpose duality is assigned to mostly commercial organizations. However, it does not exclude the possibility that noncommercial entities may also use apps for promotion despite being more likely to be perceived as *having participants’ best interests at heart*. Future studies should explore in detail the impact of purpose duality on users’ perceptions and utilization of mHealth apps.

### Limitations

Although we gathered insights to understand smartphone users’ persuasion knowledge of supporting organizations, it is not possible to generalize this study’s findings to a larger population. Future research should use quantitative methods to make systematic comparisons with the standardization assumptions underlying probability statistics. Another limitation is that the findings could be dependent on interviewers’ communication skills. As we used an in-depth interview method, interview questions themselves could be leading, which could affect participants’ responses. The interview guide also included prompts for participants who did not offer much responses (eg, liking of the health app, perceptions of quality and credibility of the health app, and decision to download or use the health app). Although we only used planned follow-up questions or probes that made interview questions more specific and helped direct the participants to the central issues of the study [67,68], it is necessary to conduct a replication study using the same methods but with a different sample to confirm the trustworthiness of the findings. In particular, probing might have led to participants’ bias in thinking elaboratively about persuasion knowledge, which could have influenced the study’s findings. In addition, social desirability could affect the participants’ responses. Finally, given time and resource constraints, some thematic analysis procedures were not implemented but are highly advised for use in future qualitative work. These refer to soliciting feedback on final themes from participants and peer researchers not involved with the study and using other methods and secondary data to study the same phenomena.

### Conclusions

Smartphone users possess high levels of persuasion knowledge of supporting organizations and understand organizations’ promotional intentions, especially those of commercial companies. However, in the complex mHealth app marketplace, users’ cognitive capacity to scrutinize all relevant mHealth app cues is limited, which may result in undesirable consequences related to personal and health-sensitive information collection and management on the web by unauthorized parties. Although users understand the potential threats to their data privacy, they often *trade* their personal and health information for free, convenient, and *fun* mHealth app services. The discussion of and recommendations for the safe and ethical use and privacy of mHealth apps should continue.

## Appendix

Multimedia Appendix 1 Interview questions.

Multimedia Appendix 2 Table S1. Conceptual definitions of themes and examples of codes.

Multimedia Appendix 3 Table S2. Participant demographics.

## Abbreviations

mHealth: mobile health
PHI: personal health information
PKM: Persuasion Knowledge Model
RQ: research question
