# Supplementary material for: Smartphone Users’ Persuasion Knowledge in the Context of Consumer mHealth Apps: Qualitative Study
Source: JMIR Mhealth Uhealth. 2021 Apr 13;9(4):e16518. doi: 10.2196/16518 (PMC8080138; doi:10.2196/16518)
Supplement: Multimedia Appendix 1 [file mhealth_v9i4e16518_app1.pdf]

**Interview Questions**

***PART I. General questions about apps***

1. What kind of apps do you have on your smartphone?
    - What apps do you use most frequently, and why?
    - What apps do you use least often, and why?
    - Approximately, how many apps do you currently have on your smartphone?
2. How often do you download new apps?
3. How often do you delete apps from your smartphone?
4. How do you find apps? (I.e., how do you learn about different apps?)
5. How do you decide what apps you want to download on your phone? (i.e., why do you download apps on your smartphone?)
6. Are there particular types of apps that you think would be useful for people like you? If so, what are they and how would they be useful?
7. Is there anything else about apps that you would like to tell us that we haven't asked you?

***PART II. Specific questions about health apps***

1. When you choose a specific **health app**, what makes you **notice** it?
2. How do you decide that the **quality** of the **health app** is good?
3. How do you decide that the **health app** is **credible**?
4. What makes you **download** the **health app**?

You are probably aware that we have different organizations that sponsor mobile apps. Some of these organizations are commercial for-profit companies and corporations, such as Coca-Cola, McDonalds, Johnson & Johnson, Nike, etc. Some of the organizations are non-profit, for example, American Cancer Association or American Diabetes Association. Some organizations are governmental, such as Centers for Disease Control and Prevention and Department of Health and Human Services.

5. Does it matter to you what organization (e.g., non-for-profit organization, commercial for-profit company, governmental agency) developed and/or sponsored the **health app**? Why yes/no?
    - How does sponsorship by **a commercial organization** influence your
        - Liking of the health app
        - Perceptions of quality and credibility of the health app
        - Decision to download/use the health app
    - How does sponsorship by **a non-profit organization** influence your
        - Liking of the health app
        - Perceptions of quality and credibility of the health app
        - Decision to download/use the health app
    - How does sponsorship by **a governmental organization** influence your
        - Liking of the health app
        - Perceptions of quality and credibility of the health app
        - Decision to download/use the health app
6. If the health app is sponsored by
    - a commercial for-profit company, what do you expect this app to **LOOK** like?
    - a non-for-profit organization, what do you expect this app to **LOOK** like?
    - a governmental agency, what do you expect this app to **LOOK** like?
7. Why do you think **commercial** organizations develop/sponsor **health apps**?

8. Why do you think **non-profit** organizations develop/sponsor **health apps**?
9. Why do you think **governmental** organizations develop/sponsor **health apps**?
10. Now, imagine that you've downloaded a **health app**. If you have to provide personal information to use the health app, will it influence your
    - Liking of the health app
    - Perceptions of quality and credibility of the health app
    - Decision to continue to use the health app

    Why yes/no?
11.
    - How much personal information you would be willing to give out to use a **health app** sponsored/developed **by a commercial organization**. What types of personal information would you be willing to provide and why?
    - What about giving up such information to use a **health app** sponsored/developed **a non-profit organization**? What types of personal information would you be willing to provide and why?
    - What about giving up such information to use a **health app** sponsored/developed **a governmental organization**? What types of personal information would you be willing to provide and why?
