# Supplementary material for: Smartphone Users’ Persuasion Knowledge in the Context of Consumer mHealth Apps: Qualitative Study
Source: JMIR Mhealth Uhealth. 2021 Apr 13;9(4):e16518. doi: 10.2196/16518 (PMC8080138; doi:10.2196/16518)
Supplement: Multimedia Appendix 2 [file mhealth_v9i4e16518_app2.pdf]

Table S1. Conceptual Definitions of Themes and Examples of Codes.

Research question	Themes	Codes
RQ1: What are study participants' levels of awareness of and interest in knowing an mHealth app affiliation with a supporting organization?	Recognition of mHealth app source is secondary to app's health management functions	Promotional support is not recognized; Promotional support doesn't' matter (n = 30)[a]
RQ2: What are the differences in the nature of participants' persuasion knowledge (e.g., recognition of target and agent, understanding of agent's characteristics and capacities, self-promotion and public service purposes,) across commercial, governmental, non-profit/non-governmental organizations that support mHealth apps?	From commercial entertainment to non-commercial information<br><br>Self-promotion and public service motivations behind mHealth apps	**Commercial Organizations:** Promote products or brands; Build brand image; Sell products; CSR (Corporate Social Responsibility); Misinformation (biased or false information); Record data (personal information, app use data, consumer opinions) (n = 68)<br><br>**Non-profit Organizations:** Help people (not-for-profit); Want support groups/fundraising; Provide Information; Collect data (n = 50)<br><br>**Government Organizations:** Mostly same as non-profit organizations; Help people (public health); Provide information; Want to collect information; Control (n = 39)
RQ3: In what ways do participants' evaluations of mHealth apps' quality and credibility differ by the type of supporting mHealth app organization?	Quality does not mean credibility	**Commercial Organizations:** Low credibility; Low likability; Great quality (best for quality); Fun/Entertainment (n = 41)<br><br>**Non-profit Organizations:** High credibility; High likability; But, low OR mediocre quality; Specificity of topic (n = 40)<br><br>**Government Organizations:** High credibility; Medium likability (less popular or boring or not user friendly); High informativeness; Broad information on various topics; Low quality (n = 45)

		**All Organization Types:** Money matters (for quality); supporting organization doesn’t matter if an app is useful or good for me (n = 15)
RQ4: What are the differences in coping mechanisms, if any, that participants implement when they are informed about mHealth app support by commercial, governmental, non-profit/non-governmental organizations, especially in light of sharing personal information within such apps?	Trade-offs<br><br>It is culture. It is normal.<br>Defenses against mHealth persuasion attempts	**Willing to share:** Social media information is not perceived as personal (already public info); Convenient to use; Forced to share (app use culture – if need, will provide BUT will worry as well); Share alternative person info such as secondary email (or Fake info –ex. Fake name); Basic/general personal info + health info [but depending on how much info want to share]; Relevant info (health + general) + but consider as SCAM, if they ask some financial information; Better to share less than more (n = 61)<br><br>In general, will provide more information to non-profits than commercial organizations, worried about sharing info with commercial entities (n = 15)<br><br>Only relevant info to all supporting organizations (n = 41)<br><br>Purpose of app (purpose of organization) – Organization-cause congruency (ex. Commercial cause matter, Non-profit/government cause match) (n = 9)<br><br>**Not willing to share:** Security risk; Privacy matter/ control personal info; Ads/ junk mails; Misuse of personal info; No share personal or health info if linked to identified personal info (n = 84)

Note: a. number of code instances
