# Supplementary material for: Smartphone Users’ Persuasion Knowledge in the Context of Consumer mHealth Apps: Qualitative Study
Source: JMIR Mhealth Uhealth. 2021 Apr 13;9(4):e16518. doi: 10.2196/16518 (PMC8080138; doi:10.2196/16518)
Supplement: Multimedia Appendix 3 [file mhealth_v9i4e16518_app3.pdf]

Table S2. Participant demographics.

	Gender	Age	Race	Education
**Total**	25			
Female	16 (64%)[a]			
Male	8 (32%)			
Refused to answer	1 (4%)			
18-24		5 (20%)		
25-34		10 (40%)		
35-44		2 (8%)		
45-54		3 (12%)		
55-64		2 (8%)		
65 and over		2 (8%)		
Refused to answer		1 (4%)		
Caucasian			12 (48%)	
Asian or Pacific Islander			5 (20%)	
African American			4 (16%)	
Other			3 (12%)	
Refused to answer			1 (4%)	
Some college				4 (16%)
Associate’s degree				1 (4%)
Bachelor’s degree				7 (28%)
Master’s degree				4 (6%)
Doctorate degree				1 (4%)
Other				1 (4%)
Refused to answer				7 (28%)

Note: a. Participants, n (%)
